# Modified Taiwan Atrial Fibrillation Score for the Prediction of Incident Atrial Fibrillation

**DOI:** 10.3389/fcvm.2021.805399

**Published:** 2022-01-28

**Authors:** Jo-Nan Liao, Su-Shen Lim, Tzeng-Ji Chen, Ta-Chuan Tuan, Shih-Ann Chen, Tze-Fan Chao

**Affiliations:** ^1^Division of Cardiology, Department of Medicine, Taipei Veterans General Hospital, Taipei, Taiwan; ^2^Institute of Clinical Medicine, Cardiovascular Research Center, National Yang Ming Chiao Tung University, Taipei, Taiwan; ^3^Department of Family Medicine, Taipei Veterans General Hospital, Taipei, Taiwan; ^4^Cardiovascular Center, Taichung Veterans General Hospital, Taichung, Taiwan

**Keywords:** incident atrial fibrillation, modified Taiwan AF score, prediction, Asian population, national cohort

## Abstract

**Background:**

We have proposed the Taiwan AF score consisting of age, male sex, hypertension, heart failure, coronary artery disease, end-stage renal disease, and alcoholism to predict incident atrial fibrillation (AF) in Asian population. We hypothesized that the modified Taiwan AF score (mTaiwan AF score) excluding alcoholism remained useful for predicting new onset AF.

**Methods:**

A total of 7,220,654 subjects aged ≥ 40 years without a past history of cardiac arrhythmia were identified from a national cohort, and 438,930 incident AF occurred during a 16-year follow-up with an incidence of 0.42 per 100 person-years. The mTaiwan AF score ranging between −2 and 14 and its predictive accuracy of incident AF was analyzed.

**Results:**

The areas under the receiver operating characteristic curve (AUCs) of the mTaiwan AF scores in predicting AF are 0.861 for 1-year follow-up, 0.829 for 5-year follow-up, 0.795 for 10-year follow-up, and 0.751 for 16-year follow-up. The risk of incident AF increased from 0.05%/year for patients with a score of −2 to 6.98%/year for those having a score of 14. Patients were classified into three groups based on the tertile values of the mTaiwan AF scores—group 1 (score −2-3), group 2 (score 4-9) and group 3 (score 10-14). The annual risks of incident AF were 0.20, 1.33, and 3.36% for group 1, 2, and 3, respectively. Compared to patients in group 1, the hazard ratios of incident AF were 5.79 [95% confidence interval (CI) 3.75-7.75] for group 2 and 8.93 (95% CI 6.47-10.80) for group 3.

**Conclusions:**

We demonstrated that the mTaiwan AF score based on age and clinical comorbidities could be used to predict incident AF in Asian population.

## Background

AF is a worldwide epidemic (1) with significant effects on morbidity and mortality (2, 3). With the trend of worldwide aging, improved diagnostic tools and better public realization, the predicted prevalence of AF keeps rising substantially (4, 5), and there is no exception for Asians (2, 3, 6). Even through, the prevalence of AF might still be underestimated based on some real-world observations and device studies (5). Therefore, it is important to efficiently identify subjects with a potential risk of AF development and to employ more aggressive strategies for cardiac rhythm screening, so that a prompt diagnosis and associated interventions can be done in time. Several risk schemes are developed for the prediction of new onset AF, and most of them were developed from non-Asian population (7–9). Recently, we have proposed a clinical scheme, the Taiwan AF score, to predict the risk of incident AF based on the national cohort analysis including 7,220,654 subjects aged ≥ 40 years (10). The Taiwan AF score included age, male sex and important comorbidities [hypertension, heart failure (HF), coronary artery disease (CAD), end-stage renal disease (ESRD), and alcoholism], and is a straightforward scheme obviating the use of personal information, electrocardiogram and echocardiography data (10). However, the factor “alcoholism” is somewhat difficult to be accurately quantified, which might prevent this scheme from extensive clinical use. Therefore, the present study aims to investigate whether a modified Taiwan AF score (mTaiwan AF score) excluding “alcoholism” can be used for AF prediction in Asian population with long-term follow-up.

## Methods

### Database

This study used the “National Health Insurance Research Database (NHIRD)” provided by Health and Welfare Data Science Center (HWDC), Ministry of Health and Welfare (MOHW), Taiwan. The National Health Insurance (NHI) system is a mandatory universal health insurance program providing comprehensive medical care coverage to all Taiwanese residents. NHIRD consists of detailed data of health care from January 1st, 1996, to December 31st, 2016, from >23 million enrollees, representing >99% of Taiwan's population. In this cohort dataset, patients' original identification numbers have been encrypted to protect their privacy, and the encrypting procedure was consistent, so that linkage of the claims belonging to the same patient was feasible. Therefore, patients can be followed continuously within the NHI database. The details about Taiwan NHIRD have been reported in our previous studies (3, 10–17). The present study was approved by the Institutional Review Board at Taipei Veterans General Hospital, Taipei, Taiwan.

### Study Population

The study design was the same as our previous study (10). In general, a total of 7,220,654 patients aged ≥ 40 years without a history of cardiac arrhythmias from January 1st, 2000 to December 31st, 2000 were identified from Taiwan NHIRD. Important comorbidities of each individual were confirmed based on the International Classification of Diseases (ICD), Ninth Revision, Clinical Modification (ICD-9-CM) codes from the NHIRD. The diagnostic accuracies of important comorbidities in NHIRD have been validated (18, 19). AF was confirmed using the ICD-9-CM code (427.31) registered by the physicians responsible for the care of patients. The diagnostic accuracy of AF based on ICD-9-CM code in Taiwan NHIRD has been validated previously (20). During a 16-year follow-up, 438,930 patients had incident AF with an incidence of 0.42 per 100 person-years.

### The Modified Taiwan AF Score

The development of the original Taiwan AF score followed the TRIPOD (Transparent Reporting of a Multivariable Prediction Model for Individual Prognosis or Diagnosis) Statement (21) and the details have been described in our previous study (10). Potential variables were identified from the Cox proportional hazards modeling and forced into an initial saturated Cox proportional hazards model. An α level of 0.1 from the saturated model was used as a threshold to enter a variable predictor into a backward elimination model. β coefficients were derived from the final Cox regression model and used to calculate the score weights of each significant predictor in the multivariable Cox regression based on the method proposed by Sullivan et al. (22). The score weight for each predictor was rounded to its closest integer as the score point. Age, male sex, hypertension, HF, CAD, ESRD, and alcoholism were thus identified with different score weight and together constitute the Taiwan AF score ranging between −2 and 15 (Table 1). In the present study, we excluded alcoholism from the prediction scheme and proposed the mTaiwan AF (Table 1). The incidence of AF (%/year) after 1-year, 3-year, 5-year, 7-year, 10-year, 12-year, and 16-year follow-up for each mTaiwan AF score was calculated. Patients were classified into three groups based on the tertile values of the mTaiwan AF scores of patients who developed AF—group 1 (score −2-3), group 2 (score 4-9), and group 3 (score 10-14).

**Table 1:** Calculations of Taiwan AF score and modified Taiwan AF score.

**Variables**	Taiwan AF score^*^	**mTaiwan AF score**
**Age, years**		
40-44	−2	−2
45-49	−1	−1
50-54	0	0
55-59	1	1
60-64	2	2
65-69	3	3
70-74	4	4
75-79	5	5
≥80	8	8
Male gender	1	1
Hypertension	1	1
Heart failure	2	2
Coronary artery disease	1	1
ESRD	1	1
Alcoholism	1	-
Total score	−2-15	−2-14

*AF, atrial fibrillation; ESRD, end-stage renal disease*.

* *The calculation rule of Taiwan AF score was based on the paper by Chao et al. (10)*.

### Statistical Analysis

The incidence of AF was calculated from dividing the number of events by person-time at risk. The Kaplan-Meier method were used to plot the cumulative incidence curves of AF for different risk groups, with statistical significance examined by the log-rank test. The diagnostic accuracy of the mTaiwan AF score in the prediction of incident AF was assessed by calculating c-indexes, based on the receiver operating characteristic (ROC) curve. All statistical significances were set at *p* < 0.05 and all statistical analyses were carried out by SPSS 17.0 (SPSS Inc. USA).

## Results

**Figure 1:**
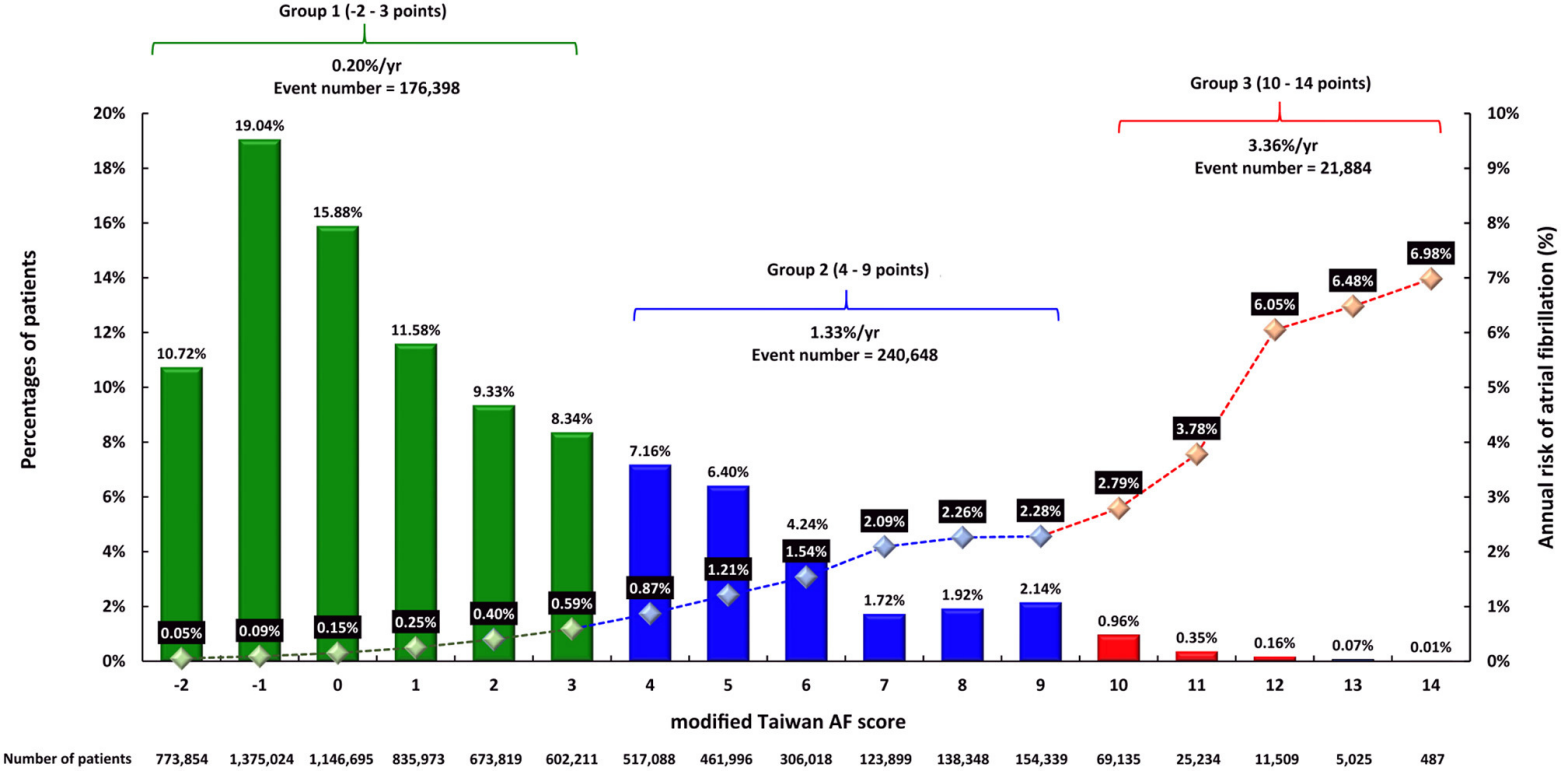
The distributions of mTaiwan AF score and the risks of incident AF during 16-year follow-up. The mTaiwan AF score ranged between −2 and 14. After a 16-year follow-up, the annual risk of incident AF increased from 0.05% for patients with a score of −2 to 6.98% for patients with a score of 14. The annual risks of incident AF were 0.20, 1.33, and 3.36% for groups 1, 2, and 3, respectively. AF, atrial fibrillation.

The distributions of each mTaiwan AF scores are shown in Figure 1. There were 5,407,576 (74.9%), 1,701,688(23.6%) and 111,390 (1.5%) patients in group 1, 2, and 3, respectively (Figure 1).

### Discrimination of mTaiwan AF Score in the Prediction of AF

**Figure 2:**
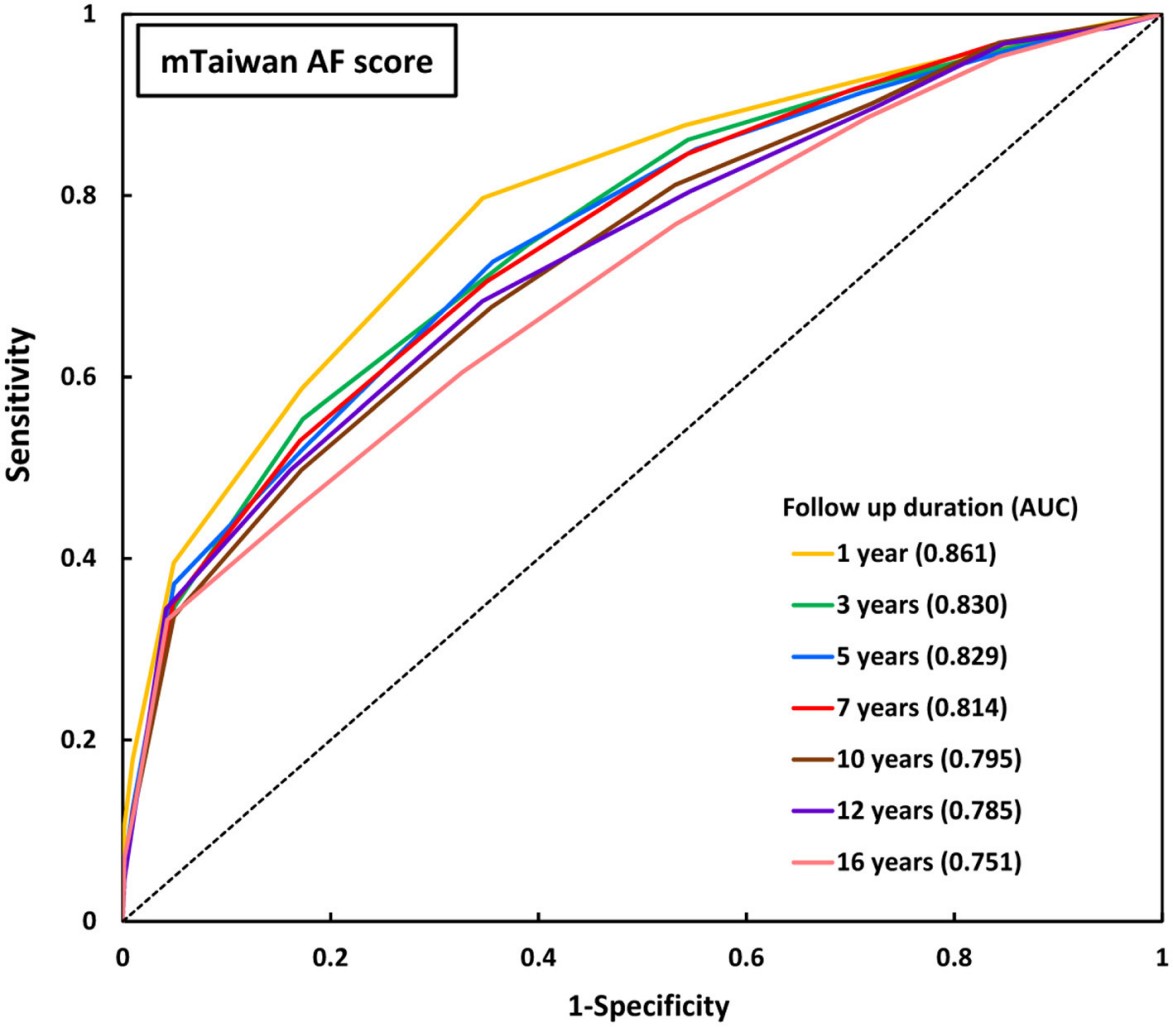
ROC curves of mTaiwan AF score in predicting incident AF. The AUCs of mTaiwan AF score ranged between 0.751 and 0.861 for different follow-up durations. AF, atrial fibrillation; AUC, area under the receiver operating characteristic curve; ROC curve, receiver operating characteristic curve.

The areas under the ROC curve (AUCs) of original Taiwan AF score and mTaiwan AF score in predicting incident AF after different follow-up durations are shown in Table 2. The AUCs of the mTaiwan AF scores are 0.861 [95% confidence interval (CI) 0.859-0.862] for 1-year follow-up, 0.829 (95% CI 0.827-0.832) for 5-year follow-up, 0.795 (95% CI 0.793-0.798) for 10-year follow-up and 0.751(95% CI 0.748-0.753) for 16-year follow-up (Figure 2).

**Table 2:** AUCs of Taiwan AF score and mTaiwan AF score in the prediction of AF after different follow-up durations.

**Follow up duration**	Taiwan AF score^*^		**mTaiwan AF score**	
	**AUC (95%CI)**	***P*-value**	**AUC (95%CI)**	***P*-value**
1 year	0.857 (0.855-0.860)	<0.001	0.861 (0.859-0.862)	<0.001
3 years	0.838 (0.837-0.840)	<0.001	0.830 (0.828-0.832)	<0.001
5 years	0.825 (0.824-0.826)	<0.001	0.829 (0.827-0.832)	<0.001
7 years	0.814 (0.813-0.815)	<0.001	0.814 (0.812-0.817)	<0.001
10 years	0.797 (0.796-0.798)	<0.001	0.795 (0.793-0.798)	<0.001
12 years	0.786 (0.785-0.787)	<0.001	0.785 (0.783-0.787)	<0.001
16 years	0.756 (0.755-0.757)	<0.001	0.751 (0.748-0.753)	<0.001

*AF, atrial fibrillation; AUC, area under the receiver operating characteristic curve; CI, confidence interval*.

* *The AUCs of Taiwan AF score in the prediction of AF were adopted from the paper by Chao et al. (10)*.

### Incidence and Risk of AF Stratified by mTaiwan AF Score

**Figure 3:**
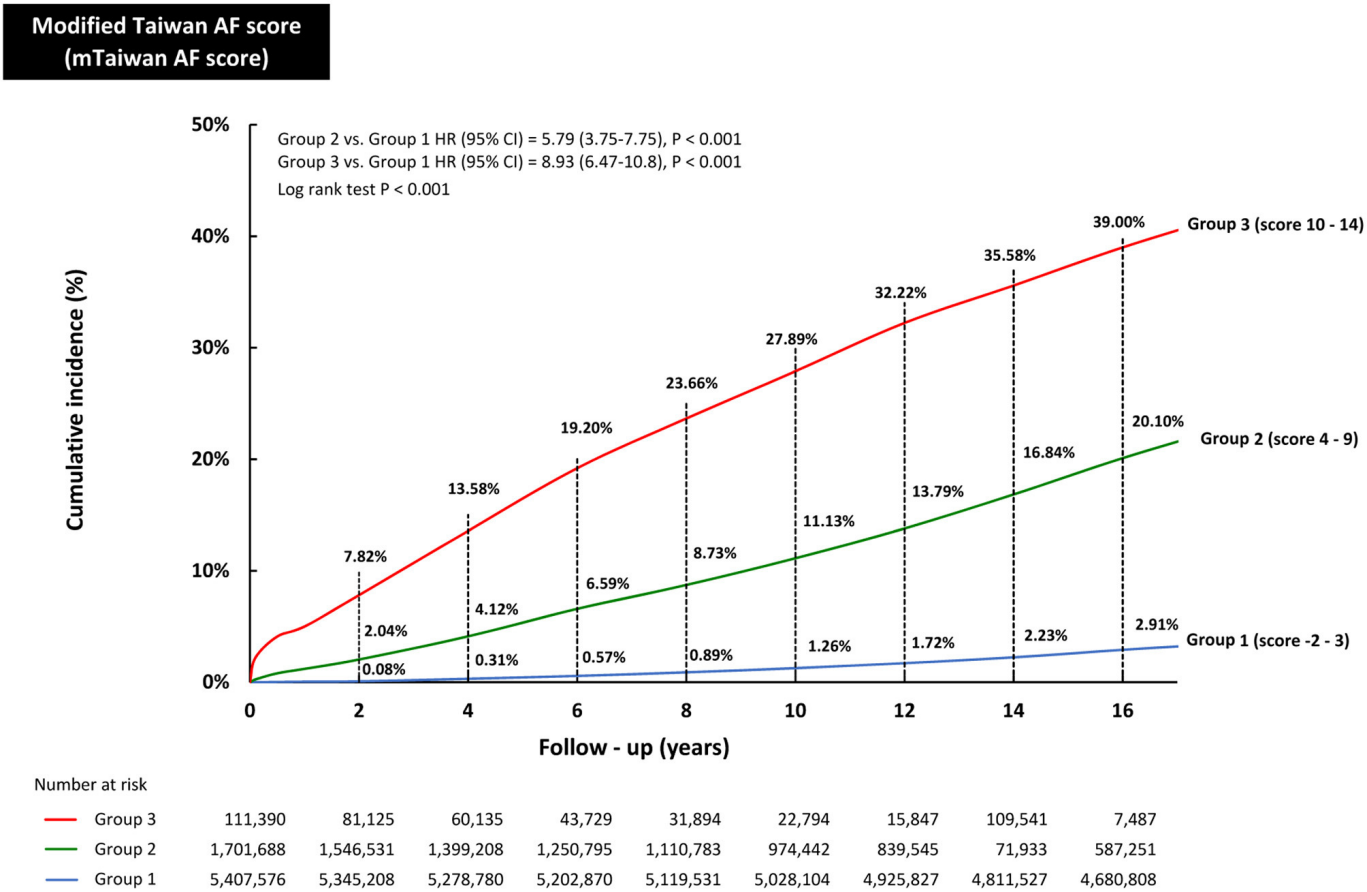
The cumulative incidence curves of developing AF in groups 1, 2, and 3. The 2-year risks of AF were 0.08, 2.04, and 7.82% for groups 1, 2, 3, respectively. The 6-year risks of AF were 0.57, 6.59, and 19.20% for groups 1, 2, 3, respectively. The 10-year risks of AF were 1.26, 11.13, and 27.89% for groups 1, 2, 3, respectively. The 16-year risks of AF were 2.91, 20.10, and 39.00% for groups 1, 2, 3, respectively. Compared to group 1, the HRs of incident AF were 5.79 (95% CI 3.75-7.75) for group 2 and 8.93 (95% CI 6.47-10.80) for group 3. AF, atrial fibrillation; CI, confidence interval; HR, hazard ratio.

The incidences of AF (%/year) of each mTaiwan AF score during different follow-up periods are shown in Table 3. After a 16-year follow-up, the risk of incident AF increased from 0.05%/year for patients with a score of −2 to 6.98%/year for those having a score of 14 (Figure 1). The annual risks of incident AF are 0.20% for group 1, 1.33% for group 2, and 3.36% for group 3, respectively (Figure 1). The cumulative incidence curves of incident AF of groups 1, 2, 3 are shown in Figure 3. The 2-year risks of AF were 0.08, 2.04, and 7.82% for groups 1, 2, 3, respectively. The 4-year risks of AF were 0.31, 4.12, and 13.58% for groups 1, 2, 3, respectively. The 10-year risks of AF were 1.26, 11.13, and 27.89% for groups 1, 2, 3, respectively. Compared to group 1, the hazard ratios (HRs) of incident AF were 5.79 (95% CI 3.75-7.75) for group 2 and 8.93 (95% CI 6.47-10.80) for group 3.

**Table 3:** Incidence of AF stratified by mTaiwan AF score after different follow-up durations.

	**Annual risk (%/year) of AF stratified by mTaiwan AF score**																
	**–2**	**–1**	**0**	**1**	**2**	**3**	**4**	**5**	**6**	**7**	**8**	**9**	**10**	**11**	**12**	**13**	**14**
1-year follow up	0.02	0.03	0.05	0.09	0.15	0.23	0.38	0.62	1.04	2.18	1.83	1.65	2.68	5.25	9.80	11.99	11.26
3-year follow up	0.02	0.04	0.06	0.12	0.20	0.33	0.55	0.81	1.20	2.05	2.08	2.12	2.85	4.55	8.05	8.97	9.36
5-year follow up	0.02	0.04	0.07	0.13	0.22	0.37	0.56	0.87	1.26	1.99	2.11	2.15	2.82	4.39	7.18	8.01	8.03
7-year follow up	0.03	0.05	0.08	0.15	0.25	0.38	0.60	0.90	1.30	1.97	2.14	2.17	2.81	4.12	6.73	7.38	7.77
10-year follow up	0.03	0.06	0.10	0.17	0.28	0.45	0.66	0.99	1.36	1.98	2.18	2.21	2.79	3.94	6.42	6.91	7.42
12-year follow up	0.04	0.06	0.11	0.19	0.31	0.50	0.72	1.06	1.42	2.04	2.21	2.24	2.79	3.89	6.22	6.72	7.38
16-year follow up	0.05	0.09	0.15	0.25	0.40	0.59	0.87	1.21	1.54	2.09	2.26	2.28	2.79	3.78	6.05	6.48	6.98

*AF, atrial fibrillation*.

## Discussion

In the present study, we proposed the mTaiwan AF score which excluded alcoholism form the original Taiwan AF score for incident AF prediction using a nationwide cohort including 7,220,654 subjects with 438,930 incident AF during a 16-year follow-up. We confirmed the usefulness of this modified scheme to predict incident AF for Asian population.

### Overview of Risk Factors of Incident AF

Risk factors for incident AF have been identified since long ago and results are somewhat variable across different studies. Potential reasons underlying the differences of reported risk factors remain unknown. Common risk factors include baseline demographics, underlying comorbidities, and cardiac structural abnormalities, such as age, male gender, obesity, hypertension, diabetes, and increased left ventricular wall thickness (23). Although the age distribution of AF differs between regions (5), the increase in AF prevalence with advancing age seems to be a worldwide phenomenon (5). Gender difference in AF incidence is also a consistent observation because a higher prevalence of AF in men than in women has been observed in most studies (5). Hypertension is the most common medical condition associated with AF worldwide, affecting 29-78% of patients with AF and there is no significant variation in the risk of AF associated with hypertension according to ethnic group (24–26). Therefore, hypertension is widely accepted to predispose individuals to AF (5), and so are CAD and HF (5, 7). Patients with ESRD had significantly increased risk of AF, which even increased farther together with other risk factors. The reported incidences of AF in patients with ESRD ranged from 1 to 14.8% depending on the co-existence of other risk factors (27). Risk factors other than age, sex, and comorbidities included race, height, personal habits, hemodynamic parameters, cardiac murmurs, electrocardiogram or echocardiographic parameters, but inconsistency remains between different stratification schemes (7–9). Nevertheless, observation from FHS and CHARG-AF scores may imply that a risk stratification scheme incorporating clinical factors but not electrocardiogram and echocardiographic parameters might provide satisfactory accuracy for predicting incident AF (7, 9).

### The Role of Alcoholism in Risk of AF Development

Alcohol consumption is ubiquitous in Western countries (28), and has been defined as light (<7 standard drinks/week), moderate (7-21 standard drinks/week), and heavy (>21 standard drinks/week) alcohol consumption, where 1 standard drink is approximately 12 g of alcohol (28). The association of alcohol and AF was first brought into attention as “holiday heart syndrome” in patients hospitalized with AF following a weekend binge (29), and more studies have been conducted on their association since then. Alcohol has complex effects to both cardiac structures and electrophysiological remodeling, and possible pathophysiological mechanisms underlying the association between alcohol and AF include direct toxicity and alcohol's contribution to obesity, sleep-disordered breathing, and hypertension (28). Alcohol could be a trigger for AF and facilitate progressive atrial remodeling with regular long-term consumption, leading to an arrhythmogenic substrate (28).

Moderate habitual consumption increases the incidence of AF in a dose-dependent but non-linearly manner, with relative risks of AF 1.08 for 7 standard drinks/week, 1.17 for 14 standard drinks/week, 1.26 for 21 standard drinks/week, 1.36 for 28 standard drinks/week, and 1.47 for 35 standard drinks/week (30–33). Heavy habitual consumptions with ≥40 standard drinks/week might be a more important risk factor than hypertension or obesity (34). Despite all the findings about alcohol and AF in real-world observations, many of the individual studies were underpowered to demonstrate a strong relationship, and some disagreements about details of alcoholism exist. For example, one meta-analysis showed that only wine and liquor, but not beer, were associated with incident AF in those consuming >14 standard drinks/week, while a community-based pooled cohort reported similar associations across different types of alcohol (33). There remains a conflict whether men and women were equally affected with alcohol consumptions (31, 35). Furthermore, most studies determined the quantity of alcohol consumption by self-reporting, rather than objective blood or urine samples, which raises the concern of precise quantification. Besides, the pattern and amount of alcohol consumption might be variable from time to time and thus it's difficult to determine the presence and degrees of habitual consumption. All conditions mentioned above highlighted the difficulty in defining “alcoholism” in clinical practice.

For easier and more extensive clinical application, we tested the accuracy of mTaiwan AF score which excluded alcoholism from the original Taiwan AF score for the prediction of incident AF in the present study. The AUCs of the mTaiwan AF score were 0.861 for 1-year follow up, 0.829 for 5-year follow up, 0.795 for 10-year follow up and 0.751 for 16-year follow up, which were quite similar to that of Taiwan AF score (0.857 for 1-year follow up, 0.825 for 5-year follow up, 0.797 for 10-year follow up, and 0.756 for 16-year follow up). Therefore, we demonstrated that the mTaiwan AF score without the consideration of alcoholism still provides reliable accuracy for the prediction of incident AF and could be easily applied in the clinical practice to replace Taiwan AF score when accurate information regarding alcoholism was not available.

### Study Limitations

In the present study, we validated the use of the mTaiwan AF score, derived from the original Taiwan AF score, in the prediction of incident AF. Since both the Taiwan AF score and mTaiwan AF score were based on the same cohort, it is expectable that the performance of mTaiwan AF score would not differ significantly from that of the original score. More studies are necessary to further validate the mTaiwan AF score in external cohorts.

## Conclusion

Based on our prior publication (10), we developed a modified clinical risk scoring scheme, the mTaiwan AF score (−2 to 14), to stratify individual risk of new-onset AF. This modified scheme is feasible and reliable for clinical assessment and can easily identify high-risk population in whom a more proactive screening strategy for AF should be taken into consideration.

## Data Availability Statement

The original contributions presented in the study are included in the article/supplementary material, further inquiries can be directed to the corresponding author/s.

## Ethics Statement

The studies involving human participants were reviewed and approved by Institutional Review Board, Taipei Veterans General Hospital. Written informed consent for participation was not required for this study in accordance with the national legislation and the institutional requirements.

## Author Contributions

J-NL is responsible for manuscript drafting. S-SL is responsible for creation of tables and figures. T-JC is responsible for the resources of database. T-CT is responsible for critical revision. S-AC is responsible for study idea and conceptualization. T-FC is responsible for study idea and organizing the whole article. All authors contributed to the article and approved the submitted version.

## Funding

This work was supported in part by grants from the Ministry of Science and Technology (MOST 107-2314-B-075-062-MY3, MOST 110-2314-B-075-059, MOST 109-2314-B-075A-011-MY3, MOST 110-2314-B-075A-014-MY3, MOST 110-2321-B-075A-001, MOST 110-2745-B-075A-001), Taipei Veterans General Hospital (V108B-015, V108B-027, V108C-090, V109C-042, V109C-186), Taichung Veterans General Hospital (TCVGH-1113-101C), Research Foundation of Cardiovascular Medicine and Szu-Yuan Research Foundation of Internal Medicine, Taipei, Taiwan. This Study was Based on Data From the Health and Welfare Data Science Center (HWDC), Ministry of Health and Welfare (MOHW), Taiwan. The Interpretation and Conclusions Contained Herein do not Represent Those of HWDC, MOHW, Taiwan.

## Conflict of Interest

The authors declare that the research was conducted in the absence of any commercial or financial relationships that could be construed as a potential conflict of interest.

## Publisher's Note

All claims expressed in this article are solely those of the authors and do not necessarily represent those of their affiliated organizations, or those of the publisher, the editors and the reviewers. Any product that may be evaluated in this article, or claim that may be made by its manufacturer, is not guaranteed or endorsed by the publisher.
